# Exercise program combined with electrophysical modalities in subjects with knee osteoarthritis: a randomised, placebo-controlled clinical trial

**DOI:** 10.1186/s12891-020-03293-3

**Published:** 2020-04-20

**Authors:** Cid André Fidelis de Paula Gomes, Fabiano Politti, Cheila de Souza Bacelar Pereira, Aron Charles Barbosa da Silva, Almir Vieira Dibai-Filho, Adriano Rodrigues de Oliveira, Daniela Aparecida Biasotto-Gonzalez

**Affiliations:** 1 Postgraduate Program in Rehabilitation Sciences, Nove de Julho University, Rua Vergueiro, 235/249, 2° Subsolo, Liberdade, São Paulo, SP CEP 01504-001 Brazil; 2 Postgraduate Program in Physical Education, Federal University of Maranhão, São Luís, MA Brazil

**Keywords:** Knee osteoarthritis, Knee pain, Exercise, Physical therapy, Modalities

## Abstract

**Background:**

It is not yet clear which of the various electrophysical modalities used in clinical practice is the one that contributes most positively when added to an exercise program in patients with knee osteoarthritis (OA). The aim of the present study was to analyze the clinical effects of the inclusion of interferential current therapy (ICT), shortwave diathermy therapy (SDT) and photobiomodulation (PHOTO) into an exercise program in patients with knee OA.

**Methods:**

This prospective, five-arm, randomised, placebo-controlled trial was carried out with blinded participants and examiners. We recruited 100 volunteers aged 40 to 80 years with knee OA. Participants were allocated into five groups: exercise, exercise + placebo, exercise + ICT, exercise + SDT, and exercise + PHOTO. The outcome measures included Western Ontario and McMaster Universities (WOMAC), numerical rating pain scale (NRPS), pressure pain threshold (PPT), self-perceived fatigue and sit-to-stand test (STST), which were evaluated before and after 24 treatment sessions at a frequency of three sessions per week.

**Results:**

In all groups, there was a significant improvement (*p* < 0.05) in all variables over time, except pressure pain threshold. We observed significant differences (*p* < 0.05) between the groups for WOMAC function (exercise vs. exercise + placebo, mean difference [MD] = 5.55, 95% confidence interval [CI] = 3.63 to 7.46; exercise vs. exercise + ICT, MD = 3.40, 95% CI = 1.46 to 5.33; exercise vs. exercise + SDT, MD = 4.75, 95% CI = 1.85 to 7.64; exercise vs. exercise + PHOTO, MD = 5.45, 95% CI = 3.12 to 7.77) and WOMAC pain, with better scores achieved by the exercise group. However, these differences were not clinically relevant when considering the minimum clinically important difference.

**Conclusion:**

The addition of ICT, SDT or PHOTO into an exercise program for individuals with knee OA is not superior to exercise performed in isolation in terms of clinical benefit. clinicaltrials.gov: NCT02636764, registered on March 29, 2014.

## Background

Osteoarthritis (OA) is a multifactorial disease related to genetic, hormonal, aging, mechanical and metabolic factors, which promote changes in focal areas causing loss of articular cartilage within synovial joints, associated with bone hypertrophy (osteophytes and subchondral bone sclerosis) and capsule thickening [1, 2]. It is one of the major causes of disability worldwide, predominantly affecting the population over 60 years old, 9.6% of men and 18% of women [3], which is only expected to increase along with increased life expectancy, overweight rates and reduced mobility of the world’s population [4].

According to the severity and level of impairment, strategies for knee OA-related interventions include surgical and non-surgical approaches [5]. Among the non-surgical, with prominent clinical use, pharmacologic interventions, which presently present a vast amount of intra-articular treatment approaches with results superior to non-steroidal anti-inflammatory drugs [6, 7]. .However, in general, pharmaceutical treatment does not promote clinically important effects in the medium- and long-term, especially in relation to improvements in pain and function [6, 8].

Supported by high-quality evidence of beneficial effects in the medium- and long-term, exercise therapy is currently indicated as the main intervention for individuals with knee OA [9]. Over an 8-week intervention, exercise therapy was found to significantly reduce pain and promote improved function and quality of life [9]. These gains are sustained from 2 to at least 6 months after cessation of treatment [10], and exercises aimed at increasing quadriceps muscle strength [11], flexibility and aerobic capacity are highlighted in the management of individuals diagnosed with lower limb OA [12].

In addition to this outstanding first-line treatment, so-called passive resources are used to assist in the management of individuals diagnosed with knee OA [13]. Among these, physical agents are widely used, including electrical, electromagnetic and phototherapeutic treatments [13]. With an emphasis on transcutaneous electrical nerve stimulation, interferential current therapy (ICT) [14, 15], shortwave diathermy therapy (SDT) [16] and photobiomodulation (PHOTO) [17] have been shown to improve pain and function, as well as increase the strength of the knee extensors [14–17].

Several recently published studies have investigated different therapies for the management of knee OA. However, it is not yet known which physical resources, routinely used in clinical practice, promote the greatest improvement in clinical variables when incorporated into exercise therapy. As OA is a globally prevalent and complex clinical condition, the more therapeutic resources found to be effective in complementing the effects of exercise therapy, the better the multimodal strategies available to resolve or reduce the signs and symptoms of knee OA.

The aim of the present study was to analyze the clinical effects of the inclusion of incorporating ICT, SDT and PHOTO into an exercise program in patients with knee OA. We tested the hypothesis that the addition of electrophysical agents would provide greater improvements then exercise therapy alone.

## Methods

### Ethical considerations

Eligible participants received full information on the objectives and procedures to be performed in the study, and those who agreed to participate signed a statement of informed consent, in accordance with the Declaration of Helsinki, 1975 and Resolution 466/12 of the National Health Council. This study received approval from the local institutional review board (process number 51391715.1.0000.5511) and is registered with ClinicalTrials.gov (NCT02636764).

### Design

The methods of the present study were established through multimodal studies previously performed by our research group [18, 19]. This was a prospective, five-arm, randomized, placebo-controlled trial with blinded participants and examiners, conducted from December 2016 to February 2018 at two physiotherapy clinics located in the city of São Paulo (SP, Brazil).

To fit the participants in the eligibility criteria, five physiotherapists with previous experience in the assessment and treatment of patients with OA performed structured evaluations in the form of an interview, in addition to performing a physical examination and considering the medical history.

In general, they underwent evaluations to attest to the eligibility criteria for participation in the study. The evaluations were structured by an interview characterized by the report of the detailed medical history and physical examination. After the evaluations, the individuals characterized as eligible to participate in the study were randomly allocated, through a randomization process, in only one of the five possible groups: exercise, exercise + placebo, exercise + ICT, exercise + SDT, or exercise + PHOTO. The hidden allocation was made using sequentially numbered opaque envelopes. This process was carried out in full, by a researcher who had not previously participated in the recruitment process of the participants, not even in the evaluation processes or in the application of the respective therapeutic resources used in each group. To carry out the study, each group was composed of two physical therapists. Totaling 10 physiotherapists with at least 5 years of experience in the management of knee OA and who still participated over 4 months in training aimed at using electrophysical modalities and performing therapeutic exercises. On the initial day of treatment, the physiotherapist opened the envelope determining the allocation of the participant. Before and at the end of the 24 treatment sessions, a blind examiner performed the evaluation procedures. Participants were informed that therapeutic exercises that could or may not involve therapy considered placebo would be employed. Thus, the participants were blinded as to the performance of the procedures involving electrophysical modalities, as they did not have a real understanding of whether the device used was really active. The methodology used in the research was structured in the norms established by the CONSORT Statement.

### Participants

All participants in this study were recruited from the waiting lists of two physiotherapy clinics and five basic health units in the state of São Paulo. Participants of both genders, aged 40 to 80 years, who had knee pain in the last 6 months and confirmed diagnosis for unilateral knee OA, according to the criteria established by the American College of Rheumatology, were included in the study. With radiographic confirmation of the diagnosis, and classified as grade 2 or 3 of the Kellgren-Lawrence Classification [20]. The diagnosis of knee OA was made through examination and the written opinion of a specialist in rheumatic diseases.

The exclusion criteria used were: history of knee trauma; signs of hip OA; lameness or use of any walking assist device; neurological disorder characterized as sensitive or motor; diagnosis of cancer, diabetes or any adverse health condition characterized as acute; cognitive impairment or psychological disorder and cardiopulmonary disease that could compromise the performance of the therapeutic exercises used in this research.

During the course of the study, none of the participants undertook any form of physical therapy, in addition to the one stipulated and defined by the randomization process of the research. In addition, they did not use intra-articular, anti-inflammatory or chondroprotective corticosteroids. The use of medications for concomitant systemic diseases (hyperglycemia, hypertension, etc.) was not controlled.

### Exercise

Exercises commonly used in clinical practice and supported by the findings of previous studies [10, 21, 22] were performed to enhance muscle strength (mainly the gluteus maximus, gluteus medius and quadriceps). All procedures for the definition and use of loads, repetitions and implementation of loads over time were based on a study by Paula Gomes et al. [19]

Aiming at the adequacy and consequent standardization of the load used to perform the exercises, 70% of a maximum painless repetition was instituted for each participant [23]. For this, the maximum load was defined before the first treatment session and, when necessary, reviewed at the end of each week. The Borg Assessment Relative Effort Scale (0 to 10 points) was used as a reference for monitoring and adjusting the load, in which 1 kg was added to the initial load when the research participant attested a score between 0 (not at all difficult) up to 4 points (somewhat difficult). For the exercises involving elastic resistance, the load was determined individually, with 10 repetitions of the exercise without pain. The elastic bands used had 8 levels of resistance divided by colors, in which the more intense coloring indicated greater resistance [23]. The sessions were held three times a week, over 8 weeks (24 sessions), on alternate days, lasting approximately 90 min each treatment session. The exercise program was as follows:

- warm up on a treadmill for 10 min with no change in grade and adopting a standardised velocity between 1.1 and 1.2 m/s [24];
- supine bridge, five sets of 30 s;
- straight leg raise in supine position, two sets of 20 repetitions;
- seated knee extension (90° to 45° of knee flexion), two sets of 20 repetitions;
- prone knee flexion, two sets of 20 repetitions;
- wall squat (0° to 60° of knee flexion), two sets of 20 repetitions with 5-s isometric contraction;
- hip abduction/lateral rotation/extension in side-lying position, two sets of 20 repetitions with 5-s isometric contraction;
- hip abduction in standing position two sets of 20 repetitions with 5-s isometric contraction; and
- hip extension/lateral rotation in prone position, two sets of 20 repetitions with 5-s isometric contraction.

### Exercise plus placebo

At the end of the exercise protocol intervention, an ultrasound device (Sonophays, EUS-0503; KLD Biosistemas Equipamentos Eletronics Ltda, Amparo, São Paulo.) was used to perform the placebo therapy. The therapy was considered a placebo as the device was turned on (so that participants could see the lights flashing on the device), but no dosing was applied to the device. For this, the individual was asked to lie supine on a stretcher, performing knee flexion of the affected leg. Slow circular movements of the transducer head were applied over the knee using transducer gel for 20 min per session.

### Exercise plus interferential current therapy

At the end of each exercise session, participants in this group received ICT using an ICT device (Sonophays, EUS-0503; KLD Biosistemas Equipamentos Eletronics Ltda, Amparo, São Paulo.). Four electrodes (8 × 6 cm) were placed around the affected knee joint. The intensity adopted by the stimulator was kept at a level considered strong, but comfortable, throughout the treatment time [25]. ICT was performed using a premodulated tetrapolar method with a carrier frequency of 4 KHz, 1/1 s sweep mode, 75-Hz frequency modulation amplitude (FMA), 25-Hz delta FMA, and automatic vector mode for 40 min. The parameters chosen for use are routinely used by our group for interventions involving knee OA analgesia.

### Exercise plus short-wave therapy

In addition to performing the exercise protocol described above, individuals allocated to the exercise + SDT group underwent SDT. A thermopulse (Ibramed, Amparo, SP, Brazil) device set to continuous mode, 27.12-MHz frequency and 150-W input was used for 20 min, and the intensity was defined based on each participant reporting a warm sensation (one sensation, described as soft but pleasant heat). For SDT application, a standard size malleable electrode (16 × 20 cm) was applied to the anterior area of the thigh, 5 cm above the upper border of the patella, and a second electrode was applied on the posterior area of the leg. For this, the participant lay supine and the knee was kept in semi-flexion at 20° [26].

### Exercise plus photobiomodulation

Prior to the exercise protocols, participants in the exercise + PHOTO group underwent photobiomodulation therapy using a laserpulse device (Ibramed, Amparo, SP, Brazil). The power of each infrared laser was as follows: wavelength of 904 nm, frequency of 9500 Hz, pulse duration of 60 ns, peak power of 70 W, average power of 0.04 W, energy density of 6 J/cm^2^ (for point), and spot size of 0.13090 cm^2^. Treatment was administered in contact with the skin using a dose of 6 J/cm^2^ applied on eight points, with a total dose of 48 J/cm^2^, each session. The eight points were: 1. the medial and lateral epicondyle of the tibia and femur, 2. the medial and lateral knee joint gap, 3. the medial edge of the tendon of the biceps femoris muscle and semitendinosus muscle in the popliteal ditch [27].

### Outcome measures

The primary outcome was physical function (subscale of WOMAC). The defined secondary outcomes were: joint pain and stiffness (subscales of WOMAC), pain intensity measured by NRPS, PPT through the use of a digital algometer, self-perceived fatigue determined by question F2.2 of the World Health Organization Quality questionnaire of Life (WHOQOL) and functionality through the sit-to-stand test (STST).

Translated and adapted from Brazilian Portuguese, WOMAC is characterized as a specific index for assessing pain, joint stiffness and physical function for individuals diagnosed with knee and / or hip OA 30. Comprised of 3 subscales, containing a total of 24 items: five on pain (score range 0 to 20), two on stiffness (score range 0 to 8), and 17 on physical functioning (score range 0 to 68), where each item received a score between 0 to 4: none = 0, low = 1, moderate = 2, severe = 3 and very severe = 4. The perception of each item was based on the 72 h prior to the assessment [28]. As a reference for the minimum clinically important difference, a variation greater than or equal to 20% of the total score was used [29].

The Numerical Rating Pain Scale (NRPS) has been adapted to different cultures and languages, characterized by its easy understanding and minimal difficulty in application. It is routinely used to assess perceived pain intensity according to the following scale: 0 (no pain) to 10 (worst possible pain) [30]. As a reference for scoring, participants assigned the score based on the last 7 days. The minimum clinically important difference of 2 points was used as a reference [31]. To evaluate the PPT, a digital algometer (DD-200; Instrutherm, São Paulo, SP, Brazil) was used. For this, the participant was instructed to position himself in a lateral position on a stretcher, in which the following points were marked on the knee to be evaluated: point 1, located 2 cm below the medial edge of the patella; and point 2, located 2 cm below the lateral edge of the patella. In this way the algometer was positioned perpendicularly at each predetermined point, a gradual pressure was applied at a constant rate of approximately 0.5 kg / cm^2 / s^ until the presence of pain was reported. The resulting PPT value was recorded in kg / cm [2]. Thus, this procedure was repeated three times at each point, with the mean value of each point being considered as a result for analysis. Although this method of analysis has good reliability [32], only one trained evaluator participated in the PPT measurements. The minimum clinically important difference was considered to be 1.62 kg / cm^2^ [33].

Self-perceived fatigue, assessed using WHOQOL-100 question F2.2: “how easily do you get tired?”. The question was scored on a scale from 1 (not at all) to 5 (extremely). This scale has been adapted for different cultures and languages, including Brazilian Portuguese, with good internal consistency, discriminant validity, criterion validity, concurrent validity and reliability [34].

To evaluate the participants’ functionality, the STST was performed. Individuals were asked to rise from seated to a standing position five times as quickly as possible, performed twice using the same bench [35]. One practice repetition was performed before the STST began. The 5-repetition STST has been examined and reported to be adequate for use in the elderly population [36].

### Sample size calculation

The sample size was calculated using Ene software (version 3.0; Autonomous University of Barcelona, Spain) and based on a clinical trial conducted by Gundog et al. [37] The WOMAC function score was chosen as the primary outcome variable. The sample size was established according to the difference of 13.6 points between groups and standard deviation of 11.4 points. Considering an 80% test power and 5% alpha, a total of 20 individuals per group was determined.

### Data analysis

For the statistical analysis, SPSS software (version 17.0; Chicago, IL, USA), was used, with a 5% significance level established for all comparisons. Intention-to-treat analysis was adopted. Histograms were created to test data normality, and all outcomes were confirmed to have normal distributions. The data were expressed as mean and standard deviation (SD) values. Mixed linear models were used using group, time and group-by-time interaction terms to calculate the adjusted mean differences between groups (MD) and 95% confidence intervals (CI).

## Results

**Fig. 1:**
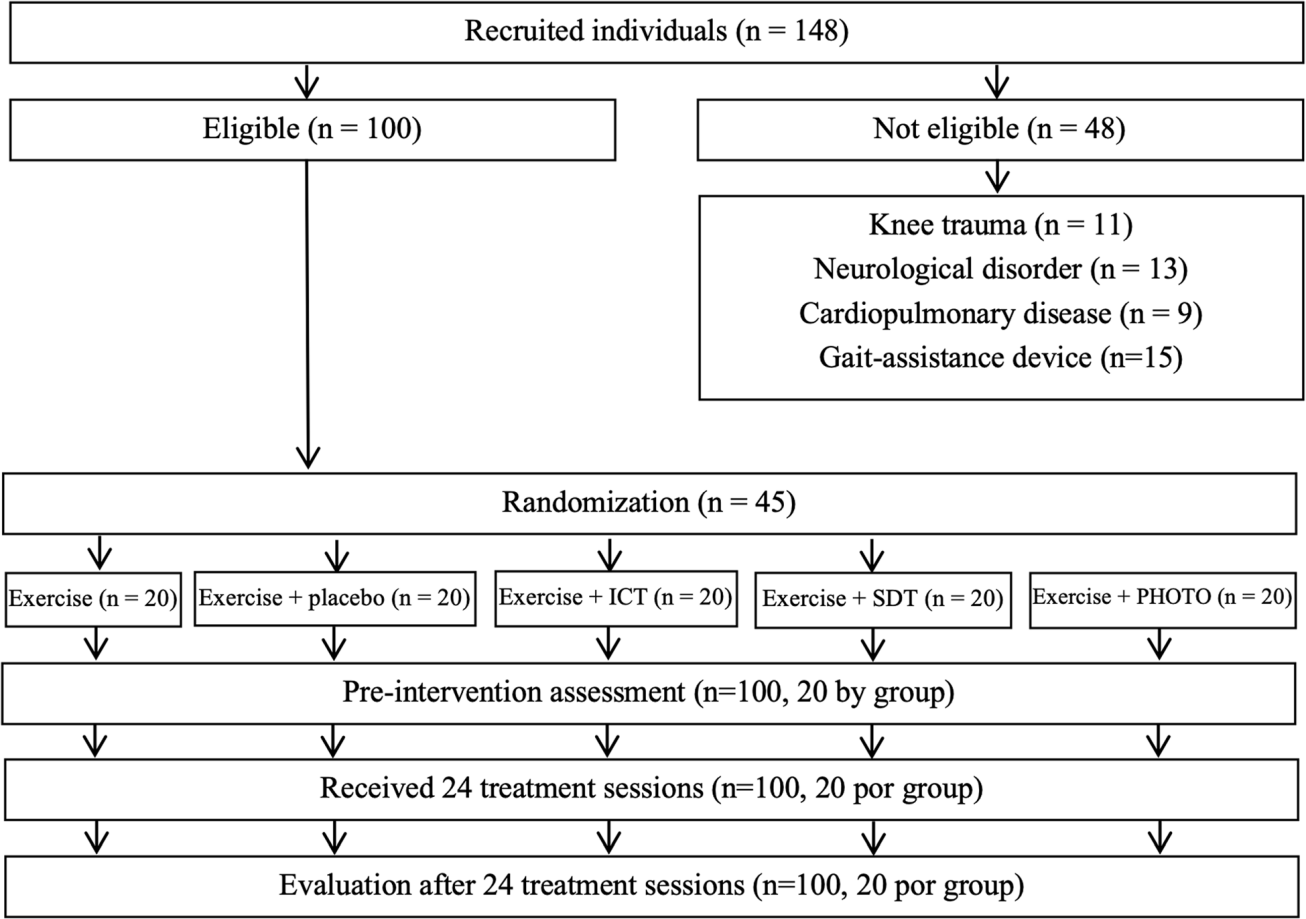
Flowchart of the study. ICT, interferential current therapy; SDT, shortwave diathermy therapy; PHOTO, photobiomodulation

To carry out the study, 148 participants were recruited. Of these, 48 were excluded for different reasons (Fig. 1). Among the 100 remaining participants, none discontinued the intervention (dropout rate of 0%). Table 1 displays the demographic characteristics of the participants included in the present study and evidence the similarity between the groups. The second column of Table 3 displays the baseline values for the outcome measures.

**Table 1:** Personal and clinical characteristics of the participants

**Characteristics**	**Exercise**	**Exercise + placebo**	**Exercise + ICT**	**Exercise + SDT**	**Exercise + PHOTO**	***p*value**
Age (years)	67.85 (4.49)	69.4 (4.45)	71.85 (2.62)	68.45 (4.62)	65.75 (4.48)	0.501
Sex (female)	17 (85%)	18 (90%)	18 (90%)	19 (95%)	20 (100%)	0.251
Body mass (kg)	69.45 (5.68)	69.90 (3.80)	71.85 (2.62)	70.9 (6.62)	69.6 (4.88)	0.241
Height (m)	1.68 (0.05)	1.67 (0.04)	1.67 (0.05)	1.65 (0.07)	1.67 (0.06)	0.413
Affected side (right)	17 (85%)	14 (70%)	15 (75%)	19 (95%)	12 (60%)	0.083

Data are expressed as mean (standard deviation) or number (percentage). ICT, interferential current therapy; SDT, shortwave diathermy therapy; PHOTO, photobiomodulation. No significant difference (*p* < 0.05, ANOVA one-way or chi-square)

Table 2 presents comparisons over time for each group. In all groups, there was a significant increase (*p* < 0.05) in all variables except the PPT. Regarding the most important analyses of the study, Table 3 presents the comparisons between the groups. We observed significant differences (*p* < 0.05) between the groups, with the exercise group showing the greatest improvement in the variables WOMAC pain and WOMAC function; however, these differences were not clinically relevant when considering the minimum clinically important difference, and should therefore be disregarded. Other similar statistical results were also found, but none were clinically important.

**Table 2:** Comparison over time of the interventions proposed in the study

**Group**	**Outcomes**	**Pre-intervention**^**a**^	**Post-intervention**^**a**^	**Mean difference**^**b**^
Exercise	WOMAC pain (score)	14.90 (1.86)	9.00 (1.41)	5.90 (5.22, 6.57) ^c^
	WOMAC stiffness (score)	6.40 (0.99)	4.35 (1.03)	2.05 (1.72, 2.37) ^c^
	WOMAC function (score)	53.70 (5.24)	38.90 (3.72)	14.80 (13.05, 16.54) ^c^
	NRPS (score)	6.55 (1.09)	4.25 (0.78)	2.30 (1.89, 2.70) ^c^
	PPT point 1 (kg/cm^2^)	2.26 (0.75)	2.36 (0.42)	−0.10 (− 0.41, 0.20)
	PPT point 2 (kg/cm^2^)	2.13 (0.63)	2.34 (0.62)	−0.20 (− 0.54, 0.12)
	Fatigue (score)	3.05 (0.60)	2.15 (0.58)	0.90 (0.50, 1.29) ^c^
	SST (score)	11.78 (1.08)	10.98 (1.26)	0.79 (0.49, 1.10) ^c^
Exercise + placebo	WOMAC pain (score)	15.30 (1.49)	10.90 (1.55)	4.40 (3.61, 5.18) ^c^
	WOMAC stiffness (score)	6.10 (0.91)	3.95 (0.82)	2.15 (1.66, 2.63) ^c^
	WOMAC function (score)	50.60 (2.92)	41.35 (2.96)	9.25 (8.21, 10.28) ^c^
	NRPS (score)	6.50 (0.68)	4.10 (0.85)	2.40 (1.91, 2.84) ^c^
	PPT point 1 (kg/cm^2^)	2.22 (0.67)	2.24 (0.75)	−0.02 (−0.50, 0.46)
	PPT point 2 (kg/cm^2^)	2.31 (0.57)	2.26 (0.46)	0.04 (−0.35, 0.44)
	Fatigue (score)	3.05 (0.68)	2.05 (0.75)	1.00 (0.52, 1.48) ^c^
	SST (score)	11.66 (0.83)	10.97 (0.66)	0.68 (0.50, 0.86) ^c^
Exercise + ICT	WOMAC pain (score)	14.75 (1.61)	11.00 (1.16)	3.75 (3.09, 4.40) ^c^
	WOMAC stiffness (score)	5.95 (0.75)	4.10 (0.44)	1.85 (1.46, 2.23) ^c^
	WOMAC function (score)	47.60 (3.76)	36.20 (3.41)	11.40 (10.43, 12.36) ^c^
	NRPS (score)	6.65 (0.98)	4.15 (0.81)	2.50 (2.11, 2.88) ^c^
	PPT point 1 (kg/cm^2^)	2.20 (0.63)	2.13 (0.37)	0.07 (−0.26, 0.41)
	PPT point 2 (kg/cm^2^)	2.37 (0.63)	2.04 (0.47)	0.33 (−0.03, 0.69)
	Fatigue (score)	3.00 (0.65)	2.05 (0.60)	0.95 (0.51, 1.39) ^c^
	SST (score)	11.15 (1.27)	10.58 (1.08)	0.56 (0.22, 0.91) ^c^
Exercise + SDT	WOMAC pain (score)	15.20 (1.15)	11.30 (1.41)	3.90 (3.19, 4.61) ^c^
	WOMAC stiffness (score)	5.75 (0.91)	3.90 (0.55)	1.85 (1.50, 2.20) ^c^
	WOMAC function (score)	46.90 (3.27)	36.85 (2.28)	10.05 (8.23, 11.87) ^c^
	NRPS (score)	6.40 (0.99)	4.40 (0.75)	2.00 (1.60, 2.40) ^c^
	PPT point 1 (kg/cm^2^)	2.20 (0.48)	2.13 (0.39)	0.07 (−0.22, 0.36)
	PPT point 2 (kg/cm^2^)	2.10 (0.46)	2.01 (0.34)	0.09 (−0.15, 0.32)
	Fatigue (score)	3.25 (0.55)	2.35 (0.59)	0.90 (0.60, 1.20) ^c^
	SST (score)	11.03 (0.87)	10.17 (0.78)	0.86 (0.74, 0.99) ^c^
Exercise + PHOTO	WOMAC pain (score)	14.00 (1.49)	10.45 (1.05)	3.55 (3.16, 3.93) ^c^
	WOMAC stiffness (score)	5.90 (0.97)	3.60 (0.60)	2.30 (1.92, 2.67) ^c^
	WOMAC function (score)	48.50 (3.23)	39.20 (2.12)	9.35 (8.01, 10.69) ^c^
	NRPS (score)	6.70 (0.86)	4.20 (1.00)	2.50 (2.03, 2.97) ^c^
	PPT point 1 (kg/cm^2^)	2.38 (0.29)	2.27 (0.34)	0.11 (−0.09, 0.30)
	PPT point 2 (kg/cm^2^)	2.06 (0.41)	2.25 (0.34)	−0.18 (− 0.43, 0.06)
	Fatigue (score)	3.10 (0.55)	2.10 (0.55)	1.00 (0.70, 1.30) ^c^
	SST (score)	10.27 (0.89)	10.27 (0.62)	0.77 (0.41, 1.12) ^c^

ICT, interferential current therapy; SDT, shortwave diathermy therapy; PHOTO, photobiomodulation; WOMAC, Western Ontario and McMaster Universities Questionnaire; NRPS, numerical rating pain scale; PPT, pressure pain threshold; SST, sit-to-stand test

^a^ Values expressed as mean (standard deviation); ^b^ Values expressed as mean difference (95% confidence interval); ^c^ Statistically significant (p < 0.05)

**Table 3:** Comparison of outcomes between the groups

**Outcome**	**Comparison**	**Mean difference (95% CI)**
WOMAC pain (score)	Exercise – Exercise + placebo	1.50 (0.34, 2.65) ^a^
	Exercise – Exercise + ICT	2.15 (1.17, 3.12) ^a^
	Exercise – Exercise + SDT	2.00 (0.89, 3.10) ^a^
	Exercise – Exercise + PHOTO	2.35 (1.63, 3.06) ^a^
	Exercise + placebo – Exercise + ICT	0.65 (0.40, 1.70)
	Exercise + placebo – Exercise + SDT	0.50 (−0.60, 1.60)
	Exercise + placebo – Exercise + PHOTO	0.85 (−0.06, 1.76)
	Exercise + ICT – Exercise + SDT	−0.15 (−1.27, 0.97)
	Exercise + ICT – Exercise + PHOTO	0.20 (−0.45, 0.85)
	Exercise + SDT – Exercise + PHOTO	0.35 (−0.60, 1.30)
WOMAC stiffness (score)	Exercise – Exercise + placebo	−0.10 (− 0.62, 0.42)
	Exercise – Exercise + ICT	0.20 (−0.33, 0.73)
	Exercise – Exercise + SDT	0.20 (− 0.27, 0.67)
	Exercise – Exercise + PHOTO	−0.25 (− 0.79, 0.29)
	Exercise + placebo – Exercise + ICT	0.30 (−0.20, 0.80)
	Exercise + placebo – Exercise + SDT	0.30 (−0.32, 0.92)
	Exercise + placebo – Exercise + PHOTO	−0.15 (− 0.78, 0.48)
	Exercise + ICT – Exercise + SDT	0.00 (−0.54, 0.54)
	Exercise + ICT – Exercise + PHOTO	−0.45 (− 0.86, − 0.03) ^a^
	Exercise + SDT – Exercise + PHOTO	− 0.45 (0.98, 0.08)
WOMAC function (score)	Exercise – Exercise + placebo	5.55 (3.63, 7.46) ^a^
	Exercise – Exercise + ICT	3.40 (1.46, 5.33) ^a^
	Exercise – Exercise + SDT	4.75 (1.85, 7.64) ^a^
	Exercise – Exercise + PHOTO	5.45 (3.12, 7.77) ^a^
	Exercise + placebo – Exercise + ICT	−2.15 (−3.63, − 0.66) ^a^
	Exercise + placebo – Exercise + SDT	−0.80 (− 2.84, 1.24)
	Exercise + placebo – Exercise + PHOTO	−0.10 (− 2.07, 1.87)
	Exercise + ICT – Exercise + SDT	1.35 (− 0.97, 3.67)
	Exercise + ICT – Exercise + PHOTO	2.05 (0.77, 3.32) ^a^
	Exercise + SDT – Exercise + PHOTO	0.70 (−1.84, 3.24)
NRPS (score)	Exercise – Exercise + placebo	−0.10 (− 0.72, 0.52)
	Exercise – Exercise + ICT	−0.20 (− 0.71, 0.31)
	Exercise – Exercise + SDT	0.30 (−0.20, 0.80)
	Exercise – Exercise + PHOTO	−0.20 (− 0.73, 0.33)
	Exercise + placebo – Exercise + ICT	−0.10 (− 0.64, 0.44)
	Exercise + placebo – Exercise + SDT	0.40 (−0.11, 0.91)
	Exercise + placebo – Exercise + PHOTO	−0.10 (− 0.68, 0.48)
	Exercise + ICT – Exercise + SDT	0.50 (− 0.07, 1.07)
	Exercise + ICT – Exercise + PHOTO	0.00 (−0.52, 0.52)
	Exercise + SDT – Exercise + PHOTO	−0.50 (−1.17, 0.17)
PPT point 1 (kg/cm^2^)	Exercise – Exercise + placebo	− 0.08 (− 0.59, 0.42)
	Exercise – Exercise + ICT	−0.17 (− 0.65, 0.29)
	Exercise – Exercise + SDT	−0.17 (− 0.58, 0.23)
	Exercise – Exercise + PHOTO	−0.21 (− 0.55, 0.12)
	Exercise + placebo – Exercise + ICT	−0.09 (− 0.53, 0.34)
	Exercise + placebo – Exercise + SDT	−0.09 (− 0.71, 0.53)
	Exercise + placebo – Exercise + PHOTO	−0.12 (− 0.56, 0.30)
	Exercise + ICT – Exercise + SDT	0.00 (−0.39, 0.40)
	Exercise + ICT – Exercise + PHOTO	−0.03 (− 0.39, 0.32)
	Exercise + SDT – Exercise + PHOTO	−0.03 (− 0.40, 0.33)
PPT point 2 (kg/cm^2^)	Exercise – Exercise + placebo	−0.25 (− 0.82, 0.31)
	Exercise – Exercise + ICT	−0.54 (−1.09, 0.00)
	Exercise – Exercise + SDT	−0.29 (− 0.75, 0.15)
	Exercise – Exercise + PHOTO	−0.02 (− 0.40, 0.35)
	Exercise + placebo – Exercise + ICT	−0.28 (− 0.85, 0.27)
	Exercise + placebo – Exercise + SDT	- 0.04 (−0.52, 0.44)
	Exercise + placebo – Exercise + PHOTO	0.22 (−0.26, 0.72)
	Exercise + ICT – Exercise + SDT	0.24 (−0.23, 0.72)
	Exercise + ICT – Exercise + PHOTO	0.51 (0.09, 0.94) ^a^
	Exercise + SDT – Exercise + PHOTO	0.27 (−0.10, 0.64)
Fatigue (score)	Exercise – Exercise + placebo	−0.10 (− 0.68, 0.48)
	Exercise – Exercise + ICT	−0.05 (− 0.60, 0.50)
	Exercise – Exercise + SDT	0.00 (−0.45, 0.45)
	Exercise – Exercise + PHOTO	−0.10 (− 0.64, 0.44)
	Exercise + placebo – Exercise + ICT	0.05 (−0.54, 0.64)
	Exercise + placebo – Exercise + SDT	0.10 (−0.46, 0.66)
	Exercise + placebo – Exercise + PHOTO	0.00 (−0.67, 0.67)
	Exercise + ICT – Exercise + SDT	0.05 (−0.48, 0.58)
	Exercise + ICT – Exercise + PHOTO	−0.05 (− 0.49, 0.39)
	Exercise + SDT – Exercise + PHOTO	−0.10 (− 0.52, 0.32)
SST (score)	Exercise – Exercise + placebo	0.11 (−0.24, 0.47)
	Exercise – Exercise + ICT	0.23 (−0.26, 0.72)
	Exercise – Exercise + SDT	−0.06 (− 0.36, 0.23)
	Exercise – Exercise + PHOTO	0.03 (−0.49, 0.56)
	Exercise + placebo – Exercise + ICT	0.12 (−0.24, 0.48)
	Exercise + placebo – Exercise + SDT	−0.18 (− 0.38, 0.02)
	Exercise + placebo – Exercise + PHOTO	−0.08 (− 0.43, 0.27)
	Exercise + ICT – Exercise + SDT	−0.30 (− 0.62, 0.02)
	Exercise + ICT – Exercise + PHOTO	−0.20 (− 0.74, 0.33)
	Exercise + SDT – Exercise + PHOTO	0.09 (−0.31, 0.51)

*CI* confidence interval, *ICT* interferential current therapy, *SDT* shortwave diathermy therapy, *LLLT* low-level laser therapy, *WOMAC* Western Ontario and McMaster Universities Questionnaire, *NRPS* numerical rating pain scale, *PPT* pressure pain threshold, *SST* sit-to-stand test. ^a^ Statistically significant (*p* < 0.05)

## Discussion

This randomised controlled trial investigated the clinical effects of incorporating ICT, SDT or PHOTO into a therapeutic exercise program for individuals with knee OA when compared to a group that received exercise alone and to a group that received exercise and ultrasound placebo therapy. Looking at the between-group comparisons in terms of the clinically important minimum difference, it is evident that the addition of ICT, SDT or PHOTO did not increase the clinical benefit after 8 weeks of treatment (primary and secondary variables) when combined with an exercise protocol for knee OA.

Regarding the use of ICT and its long-term effects, four systematic reviews with meta-analyses have been published to date. Two of these [14, 38] addressed the use of ICT in the general management of acute and chronic skeletal muscle pain, including the management of knee OA. The others addressed the use of electrical stimulation, including ICT, specifically in relation to the management of knee OA [39, 40].

Our findings are in contrast with previous published studies on ICT. Fuentes et al. (2010) [38] stated that the inclusion of ICT in a multimodal treatment program promotes pain relief in chronic musculoskeletal conditions. Almeida et al. (2018) [14] reported the effectiveness of ICT for improving pain and function analysed using the WOMAC. Zeng et al. (2015) [39] highlighted ICT as a promising treatment for relief of pain associated with knee OA. The findings of our study are similar to those of Rutjes et al. (2009) [40], who did not confirm the effectiveness of ICT for pain relief in individuals with knee OA.

The addition of SDT did not potentiate the effects of exercise therapy. Despite controversial evidence in the literature [41, 42], it is routine to prescribe SDT for the management of knee OA [43]. However, as we used different forms of pain assessment, our results show that the effects of this therapy appear to have a more limited therapeutic window than the 12 weeks post-treatment indicated by Laufer and Dar (2012) [41], and confirms the ineffectiveness of SDT for increasing functionality.

In the same way as Atamaz et al. (2012) [43], we used a continuous modality because we believe that this modality can modulate the anti-inflammatory response and reduce muscle spasms and joint stiffness. There are many questions about the variability of parameters and the choice of modalities employed in clinical trials involving physical therapy agents [41]. However, the current literature [41, 42] offers conflicting information as to the choice of modality employed.

Photobiomodulation is another resource that, despite the heterogeneity of available scientific evidence on the effectiveness of its application in knee OA, is routinely used to relieve pain and improve function [44, 45]. This heterogeneity is often attributed to the variation in doses used by different studies [46]. Therefore, we performed irradiation without the use of clusters, which would cover larger treatment areas, using a similar dose and irradiation points to those used by Hegedus et al. (2009) [27]. This previous study presented different findings to the current study, reporting a reduction in knee OA-related pain with the addition of PHOTO.

We expected the three physical resources to complement exercise therapy. They are usually associated with an improvement in pain, as seen in a previously study by our group [19]. We believe that the expected clinical benefit from the addition of physical agents was not evident in the current study due to the inability of these resources to promote improvements in synovial inflammation and cartilage degradation, as previously reported in experimental models [42, 47] and in humans [48].

The present study has some limitations that should be addressed, which also offer opportunities for future studies. First, although the study has as a strong point the number of treatment sessions (24 sessions), we did not perform follow-up after the end of treatment to determine the long-term effects of the interventions. Second, the therapists involved in carrying out the study could not be blinded to the treatments. Third, two physiotherapists performed the respective treatments per group, and despite the same level of experience and previous training, we did not analyse the reproducibility of interventions in each group. Fourth, we could not establish a control over the use of painkillers. Finally, we made available if the volunteer requested transportation to the place of care, which may have influenced adherence to treatment.

## Conclusion

The addition of ICT, SDT or PHOTO to an exercise program for individuals with knee OA does not increase the clinical benefit compared to exercise performed in isolation.

## Data Availability

Data and material related to this study is available from the corresponding author on request.

## Abbreviations

FMA: Frequency Modulation amplitude
ICT: Interferential Current Therapy
NRPS: Numerical Rating Pain Scale
OA: Osteoarthritis
PHOTO: Photobiomodulation
PPT: Pressure pain threshold
SDT: Shortwave diathermy therapy
STST: Sit-to-stand test
WOMAC: Western Ontario and McMaster Universities
WHOQOL: World Health Organization Quality of Life
